# Multicenter, dual fractionation scheme, single core lab comparison of rectal volume dose reduction following injection of two biodegradable perirectal spacers

**DOI:** 10.1002/acm2.14086

**Published:** 2023-06-28

**Authors:** Michael Kos, Rizwan Nurani, Paulo Costa, Mateuzs Dabkowski, Joana Vale Ferreira da Silva, Shawn Zimberg, John Keane

**Affiliations:** ^1^ Northern NV Radiation Oncology Spokane Washington USA; ^2^ Western Radiation Oncology Campbell California USA; ^3^ CUF Porto Instituto Rua Fonte das Sete Bicas Porto Portugal; ^4^ Onology Center – Institute of Maria Skłodowska – Curie Warsaw Poland; ^5^ Advanced Radiation Centers of New York Lake Success New York USA

**Keywords:** balloon spacer, organ at risk, perirectal spacer, prostate cancer, SBRT, ultra hypofractionation, VMAT

## Abstract

**Purpose:**

A multicenter, double‐arm, central core lab, retrospective study was performed to compare the rectal dosimetry of patients implanted with two injectable, biodegradable perirectal spacers, in conventional fractionation (CF), as well as ultrahypofractionation (UH) treatment plans.

**Methods and materials:**

Fifty‐nine patients were enrolled into the study in five centers: two centers in Europe, which implanted a biodegradable balloon spacer in a total of 24 subjects and three centers in the US, which implanted the SpaceOAR in 35 subjects. Anonymized CTs (pre and post‐implantation) were reviewed by the central core lab. For VMAT CF plans rectal V50, V60, V70, and V80 were calculated. For UH plans, a corresponding rectal V22.6, V27.1, V31.37, and V36.25 were established representing 62.5%, 75%, 87.5%, and 100% of the 36.25 Gy prescribed dose.

**Results:**

For CF VMAT, a comparison between the balloon spacer and the SpaceOAR revealed a significant difference of 33.4% decrease in mean rectal V50 (71.9% vs. 38.5%, *p* < 0.001), 27.7% in mean rectal V60 (79.6% vs. 51.9%, *p* < 0.001), 17.1% difference in mean rectal V70 (84.1% vs. 67.0%, *p* = 0.001), and a significant difference of 3.0% (*p* = 0.019) in mean rectal V80 (87.2% vs. 84.2%). With UH analysis, the mean rectal dose reduction for the balloon spacer compared to the SpaceOAR was 79.2% and 53.3% for V27.1 (*p* < 0.001), 84.1% and 68.1% for V31.71 (*p* = 0.001), and 89.7% and 84.8% for V36.25 (*p* = 0.012), respectively.

**Conclusion:**

Rectal dosimetry is more favorable for treatment with the balloon spacer compared with SpaceOAR. Further research, particularly in the context of a prospective randomized clinical trial design, is needed to assess the acute and late toxicity experience as well as physician satisfaction with achieving symmetrical implantation, and ease of use in light of increasing clinical use.

## 1 INTRODUCTION

Radiation therapy (RT) for patients diagnosed with localized prostate cancer has rapidly evolved during the past decade. However, radiation induced rectal toxicity remains a concern due to the proximity between the prostate and the rectum.^1^, ^2^ A potential solution that has been proposed in recent years is the deployment of biodegradable implantable perirectal spacers.^3^, ^4^, ^5^ Biodegradable spacers, including hyaluronic acid,^6^ absorbable hydrogel,^7^ polyethylene‐glycol‐based hydrogel,^8^ collagen implants,^9^ and saline‐filled balloons,^10^ have been used in an effort to separate the anterior wall of the rectum from the irradiated area with a variety of devices designed to increase the physical prostate‐anterior rectal wall distance. Two widely used implantable spacers are the SpaceOAR (Boston Scientific, Massachusetts, USA)^11^ and the balloon spacer, (BioProtect, Tzur Yigal, Israel).^12^ Both are designed to temporarily separate the anterior rectal wall from the prostate during radiation treatment. The spacers are implanted transperineally under TRUS guidance between the prostate and rectum, prior to the initiation of RT for prostate cancer.

Both spacers are intended to temporarily position the anterior rectal wall away from the prostate during radiotherapy for prostate cancer, while sparing the anterior rectum from excessive radiation.^3^ They are made of biodegradable material that mechanically creates a space, maintains that space for the entire course of prostate radiotherapy treatment and absorbed by the patient's body over time. Data from the literature provides evidence for these spacer's safety profile, rectal dosimetric gain, and improved quality of life outcomes relative to prostate cancer patients treated with EBRT with no spacer.^3^, ^13^

However, there are differences in how the volume between the prostate and rectal interface is created based upon the devices themselves. The implantation procedure of the balloon spacer includes establishing a working channel along the plane from prostate apex to base using a beveled tip dilator dissection with an option for hydro dissection. The balloon spacer is inflated with saline, providing about 18 mm space height,^13^ and can be deflated and repositioned if needed, both laterally and along the distal/proximal planes, for optimal uniformity of spacing. Whereas SpaceOAR is a polyethylene glycol gel that is injected as a polymer. It polymerizes in seconds and cannot be moved, providing 8.7–16.5 mm space.^3^

This multicenter, double‐arm, central core lab, retrospective study aims to compare the rectal dosimetry in patients implanted with either the SpaceOAR or the balloon spacer, in the context of CF, as well as UH treatment plans.

## 2 METHODS AND MATERIALS

### 2.1 Patients

Fifty‐nine patients were enrolled in the study in five centers: two centers in Europe, which implanted the biodegradable balloon spacer in a total of 24 patients and three centers in the USA, which implanted the SpaceOAR in 35 subjects. The study received approval by the local Ethics Committees and Institutional Review Boards. All patients underwent prostate radiotherapy with either the balloon spacer or with the SpaceOAR.

Computed tomography (CT) for planning was done before and after spacer implantation for all patients. For each patient, both CT scans were used to obtain comparable “pre and post” planning simulation using identical treatment planning and dose constraints: The clinical target volume (CTV) included the prostate with seminal vesicles, the planning target volume (PTV) was defined as the CTV with margins of 5 mm in all directions (including posterior direction), the rectum was contoured as a solid structure from the anus to the rectosigmoid flexure. Contouring of the rectum was limited, to the extent possible, to the same number of corresponding CT slices on both image sets (pre and post spacer implantation).

### 2.2 Plan generation and statistical analysis

This study utilized a single core laboratory for analysis of CT images and generation of the DVH data. All target and organ at risk (OAR) volumes were defined and contoured by the same physician. The Core‐Lab physician delineated the target volumes and critical structures on both the pre‐ and post‐implant CT datasets according to predefined guidelines to assure consistency of structure delineation between the two datasets. For each patient, a pre‐ and post‐implant treatment plans were generated using identical dose specifications. Plans were generated containing two fields assigned to the Varian TrueBeam linear accelerator and with 6MV photon energy. The isocenters for both fields are placed within the Planning Target Volume (PTV). Both fields have full arc properties. The chosen gantry rotations are as follows: 179° to 181° (counter‐clockwise) for Field 1, and 181° to 179° degrees (clockwise) for Field 2. The collimator angles used for the fields are 45° for Field 1, and 315° for Field 2 and procedures, assuming same classification of clinical stage for all patients.

Treatment plans were optimized using the RapidPlan (knowledge‐based treatment planning system; Varian, Palo Alto, California, USA) to ensure consistency, standardized treatment planning process, and minimize intra‐observer variability. The dose was calculated Varian's AAA algorithm version 15.606. Two treatment plans were used for each patient: VMAT treatment plans were generated for CF of 81 Gy in 45 fractions. The dose constraints to the rectum were generated using RapidPlan's dose volume histogram (DVH) estimation model, which is based upon each patient's individual anatomy. Treatment plans were generated for UH of 36.25 Gy in five fractions. Once again, the rectal dose constraints were generated by RapidPlan's DVH estimation model. Once the plan is normalized such that 98% of the PTV receives 100% of the prescribed dose, the dose statistics were evaluated. For VMAT CF plans rectal V50, V60, V70, and V80 were calculated. For UH plans, a corresponding rectal V22.6, V27.1, V31.37, and V36.25 were established representing 62.5%, 75%, 87.5%, and 100% of the 36.25 Gy prescribed dose.

Patient demographic and baseline characteristics between balloon spacer and SpaceOAR are summarized as mean ± standard deviation (SD) for categorical variables and compared using Wilcoxon sum rank test for not normally distribution.

The dosimetric variables considered were rectal volume, maximum, and mean dose to the rectum), and volumes (percentage and absolute) of rectum receiving V50, V60, V70, and V80 Gy CF and V22.6 , V27.1 , V31.7, and V36.25 Gy for UH. Wilcoxon sum rank test was used to compare continuous outcomes that are not normally distributed. All reported *p*‐values are two‐sided. A *p*‐value < 0.05 was considered statistically significant. The statistical analysis was carried out through SigmaPlot software (SigmaPlot version 12.5, Systat Software, San Jose, California, USA).

## 3 RESULTS

Fifty‐nine men with prostate cancer received VMAT following balloon spacer and SpaceOAR placement. Patient characteristics are described in Table 1. The mean age of patients was 71 ± 7.7 years in the balloon spacer group and 68.8 ± 6.8 years in the SpaceOAR group. All patients underwent a pretreatment prostate‐specific antigen (PSA) determination (mean 7.9 ± 5.0 and 8.3 ± 7.5 ng/mL in the balloon spacer group and SpaceOAR group, respectively). Twenty‐one (88%) men in the balloon spacer group and 35 (100%) in the SpaceOAR group were identified with low‐intermediate risk prostate cancer. Three men (12%) with high‐risk prostate cancer were identified in the balloon spacer group.

**TABLE 1:** Patient characteristics at baseline.

	Balloon Spacer	SpaceOAR	*p*‐value*
**Age (Years)**			
*n*	24	35	
Mean ± SD	71.0 ± 7.7	68.8 ± 6.8	0.30
**PSA (ng/mL)**			
*n*	24	35	
Mean ± SD	7.9 ± 5.0	8.3 ± 7.5	0.60
**Gleason**			
% with combined Gleason			0.89
5	1 (5%)	0 (0%)	
6	9 (39%)	12 (36%)	
7	9 (39%)	20 (59%)	
8‐10	4 (17%)	2 (6%)	
**Tumor stage**			
% with T stages shown			0.003
T1	8 (38%)	27 (77%)	
T2	13 (62%)	8 (24%)	

Abbreviations: PSA, prostate‐specific antigen; SD, standard deviation.

* *p*‐values are calculated using Wilcoxon matched‐paired signed‐rank test.

**FIGURE 1:**
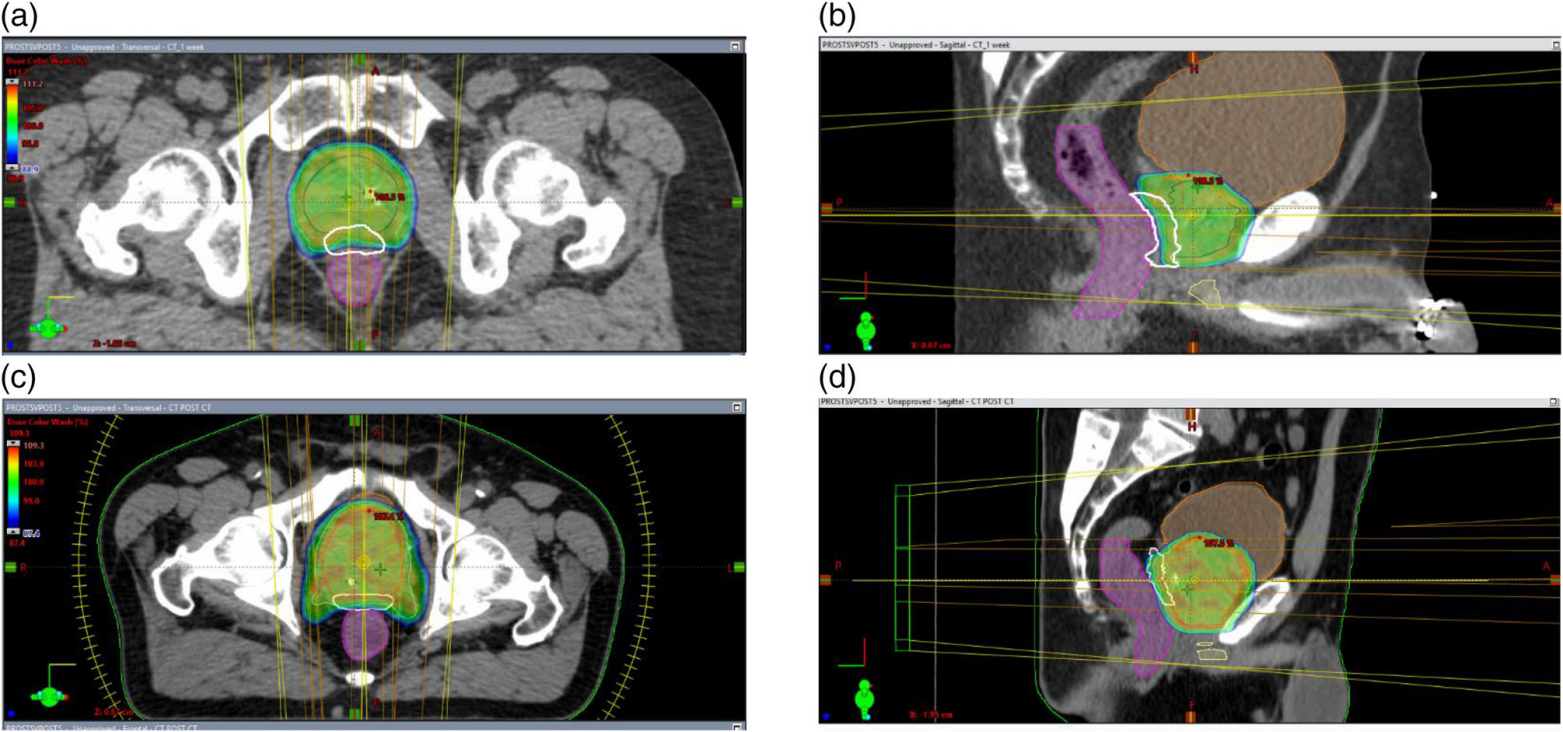
Prostate cancer VMAT treatment plan depicting simulated dose distribution in axial (a) and sagittal (b) views after balloon spacer implantation and in axial (c) and sagittal (d) views after SpaceOAR implantation. Colorwash for dose scaling was set in the range 89.0‐110%.

**FIGURE 2:**
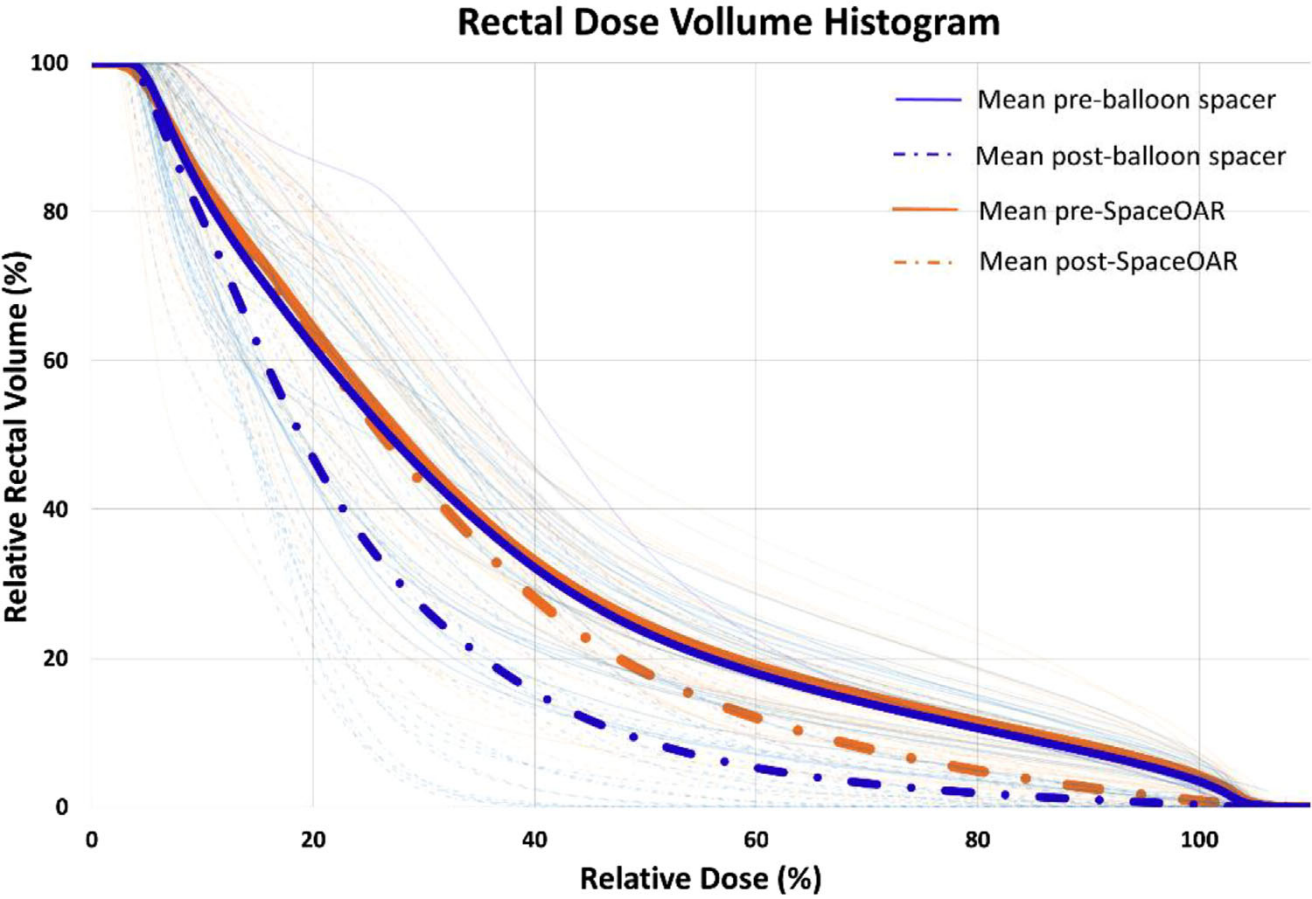
Individual rectal dose‐volume histogram (DVH) curves for hypofractionation radiation treatment at V22.6 , V27.1 , V31.7, and V36.25 Gy.

Anonymized CTs were uploaded to the central core lab (ARC, New York). Figure 1 depicts a dose distribution for prostate cancer treatment following balloon spacer and SpaceOAR implantation, in which parts of the prostate has been covered while dose decreases moving away from the PTV allowing for reduced dose to the rectum. An individual dose‐volume histograms of the rectum for each 24 BioProtect Balloon and 35 SpaceOAR subjects shown in Figure 2.

**FIGURE 3:**
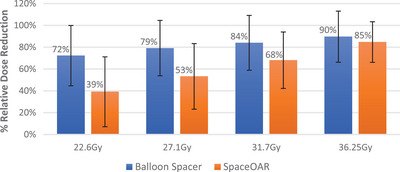
Difference, in sparing of the rectum with hypofractionation radiation treatment at V22.6 , V27.1 , V31.7, and V36.25 Gy (presented as the % relative reduction ± Standard dev.).

With UH analysis, the mean rectal dose reduction for the balloon spacer compared to the SpaceOAR was 72.3% and 39.2% for V22.6 Gy (*p* < 0.001), 79.2% and 53.3% for V27.1 (*p* < 0.001), 84.1% and 68.1% for V31.71 (*p* = 0.001), and 89.7% and 84.8% for V36.25 (*p* = 0.012), respectively. These results are graphically represented in Figure 3.

**FIGURE 4:**
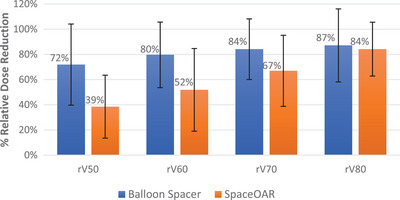
Difference in sparing of the rectum with VMAT radiation treatment at V50, V60, V70, and V80Gy (presented as the % relative reduction ± Standard dev.).

When simulated with CF for comparison (Figure 4) between the balloon spacer and the SpaceOAR, a significant 33.4% difference in rectal dose reduction was noted for V50 (71.9% vs. 38.5%, *p* < 0.001). In addition, a 28% difference was noted for V60 reduction (79.6% vs. 51.9%, *p* < 0.001) as well as a 17% difference in V70 reduction (84.1% vs. 67.0%, *p* = 0.001). Reduction of V80 was 87.2% for balloon spacer subjects, and 84.2% for SpaceOAR subjects (*p* = 0.019).

Table 2 shows the mean rectal DVH before and after spacers implantation for both balloon spacer and SpaceOAR across the two treatment radiation schemes.

**TABLE 2:** Mean ± SD rectal dose volume histogram (DVH) comparing pre and post implantation DVHs between the balloon spacer and the SpaceOAR, when applying Ultra Hypofractionation and VMAT.

	Balloon Spacer				SpaceOAR			
Target Prescribed Dose (Gy)	% Pre spacer (Mean± SD, p‐value*)	% Post Spacer (Mean± SD, p‐value**)	% of Relative Reduction (Mean± SD, p‐value***)	% of Relative Reduction (Median [range])	% Pre spacer (Mean± SD, p‐value*)	% Post Spacer (Mean± SD, p‐value*)	% of Relative Reduction (Mean± SD, p‐value***)	% of Relative Reduction (Median, [Range])
rV22.6	16.9 ± 5.7 (p=0.57)	4.78 ± 5.0 (p=0.0002)	72.3 ± 27.6 (p=0.0002)	81.2 (8.7‐100.0)	18.4 ± 6.9 (p=0.56)	10.7 ± 5.5 (p=0.0001)	39.2 ± 32.0 (p=0.0002)	40.3 (−13.3‐100.0)
rV27.1	12.2 ± 4.4 (p=0.56)	2.5 ± 3.3 (p=0.0001)	79.2 ± 25.4 (p=0.0002)	89.2 (12.5‐100.0)	13.4 ± 5.3 (p=0.56)	6.0 ± 3.8 (p=0.0001)	53.3 ± 30.1 (p=0.0002)	52.5 (−60.6‐99. 8)
rV31.7	8.2 ± 3.3 (p=0.48)	1.2 ± 2.1 (p=0.0004)	84.1 ± 25.1 (p=0.001)	96.1 (7.6‐100.0)	9.2 ±3.8 (p=0.48)	2.9 ± 2.4 (p=0.0004)	68.1 ± 25.8 (p=0.001)	70.5 (−17.3‐100.0)
rV36.25	3.4 ± 1.7 (p=0.10)	0.3 ± 0.8 (p=0.003)	89.7 ± 23.4 (p=0.011)	100.0 (5.1‐100.0)	4.2 ± 1.8 (p=0.10)	0.7 ± 1.0 (p=0.003)	84.8 ± 25.1 (p=0.011)	92.6 (41.4‐100)
rV50	15.6 ± 5.8 (p=0.30)	4.6 ± 5.4 (p=0.0002)	71.9 ± 32.2 (p=0.0002)	84.8 (−11.0‐100.0)	18.0 ± 7.1 (p=0.30)	10.5 ± 5.8 (p=0.0002)	38.5 ± 37.7 (p=0.0002)	41.2 (−4.0‐100.0)
rV60	11.5 ±4.4 (p=0.29)	2.4 ±3.4 (p=0.0001)	79.6 ±26.0 (p=0.0002)	91.3 (10.7‐100.0)	13.6 ±6.2 (p=0.29)	6.5 ±4.9 (p=0.0001)	51.9 ±32.8 (p=0.0002)	54.9 (−83.7‐99.5)
rV70	7.8 ±3.3 (p=0.24)	1.2 ±2.1 (p=0.0003)	84.1 ±24.0 (p=0.001)	96.9 (15.4‐100.0)	9.5 ±4.7 (p=0.24)	3.2 ±3.4 (p=0.0003)	67.0 ±28.2 (p=0.001)	70.7 (−35.2‐100.0)
rV80	3.5 ±2.04 (p=0.16)	0.4 ±0.8 (p=0.007)	87.2 ±29.0 (p=0.02)	100 (−24.4‐100.0)	4.4 ±2.5 (p=0.16)	0.9 ±1.6 (p=0.007)	84.2 ±21.4 (p=0.02)	91.6 (−2.7‐100.0)

Abbreviations: rV = rectal volume; SD = standard deviation.

p‐values are calculated using Wilcoxon matched‐paired signed‐rank test.

* The difference between the balloon spacer and SpaceOAR pre implantation rectal dose values.

** The difference between the balloon spacer and SpaceOAR post implantation rectal dose values.

*** The difference between balloon spacer and SpaceOAR relative rectal dose reduction.

## 4 DISCUSSION

To our knowledge, the current study is the first multicenter central core lab report comparing two biodegradable perirectal spacers in terms of their defining function: the ability to reduce radiation exposure in the rectum of patients undergoing prostate RT.

The SpaceOAR has been approved by the FDA and used successfully worldwide. Clinical trials in Europe and the USA have demonstrated that the hydrogel is safe, and that the space created significantly reduces the radiation delivered to the rectum.^14^, ^15^, ^16^, ^17^, ^18^

An earlier comparison of these two spacers^4^ was reported by a single hospital in 2019: The results described by Schorghofer et al. are consistent with the results reported herein: Mean dose to the rectum and bladder was 24.71 Gy (SD = 7.10) and 24.44 Gy (SD = 12.70) for the balloon spacer and 29.92 Gy (SD = 7.96) and 30.15 Gy (SD = 12.68) for the SpaceOAR spacer. The V50 for rectum and bladder was 8% and 18% for the balloon spacer, and 16% and 23% for the gel spacer, respectively. Furthermore, in their report, Schorghofer et al. showed a correlation (r)of dose to the rectum and the CTC (Common Terminology Criteria) GI toxicity score (*r* = 0.201, *p* < 0.05). Although our report did not evaluate GI toxicities, the dosimetric difference was similar between the two studies.

It is noteworthy that among our report, the two spacers performed similarly in the V80 reduction, and UH corresponding V36.25 reduction with ∼5% difference between the spacers, but at 87.5%, 75%, and 62.5% of the full dose (V70, V60, and V50), a significant gap emerged: relative V70 reduction was 17% higher with the balloon spacer, V60 reduction was 28% higher for the balloon spacer, and V50 reduction was 33% higher with the balloon spacer.

Our findings comparable with the first results of the French multicenter open‐label study (BioPro‐RCMI‐1505) using the balloon spacer.^13^ In this study reported by Latorzeff et al., 85.2% reduction in the mean rectal rV70 was achieved with the balloon spacer. Our findings with the gel spacer also appear to be consistent with other controlled randomized studies that have reported dose reduction to the rectum when using rectal spacers during radiation treatment in prostate cancer patients.^3^

Future long term‐controlled studies should investigate whether more complete and consistent reduction in rectal radiation translates to better long‐term clinical outcomes.

This report has several limitations: Prior spacer implantation experience is inconsistent across study arms; specifically, the experience level was consistently higher in the SpaceOAR arm, ahead of the sets of patients reported. Notwithstanding, the nonrandomized nature of our analysis precludes us from ruling out hidden advantages (unrelated to experience) that may have favored the users of the balloon spacer technology. Such advantages are possibly related to the balloon pre‐defined shape, size, and symmetry. These technological characteristics of the balloon may contribute to the significant dose reduction as shown in our study compared to the gel, as previously demonstrated by Fischer‐Valuck which evaluated the symmetry correlation to rectal dose reduction.^19^ The study is also limited in scope with an average of 12 subjects per site. Furthermore, the study was not designed to capture acute and/or long‐term patient bowel and urinary quality of life, which constitute critical clinical issues. As the study is a nonrandomized observational study, *p*‐values should be carefully interpreted.

Although baseline DVH values tended slightly to be higher in the SpaceOAR group compared to baseline DVHs values in the balloon spacer group, these differences differ insignificantly (*p* > 0.05); nevertheless, the measurement of the relative dose reduction neutralizes the small differences in the baseline values.

Given that the mean baseline characteristics were found to be similar between the two study arms, and the use of a central core lab, it is tempting to conclude that rectal dose reduction was higher among subjects receiving the balloon spacer. The superiority of the balloon, achieving greater rectal dose reduction may be attributed to the (1) larger perirectal separation provided by the balloon (typically 18 mm)^12^ compared to the SpaceOAR with a perirectal separation of 12.6 + 3.9 mm as reported in literature,^3^ (2) the ability to deflate the balloon, inflate it again and repositioned it to provide optimal spacing, and finally, the balloon enables structured and predictable spacing shape that is consistently visible during implantation, allowing proper and uniform spacer placement. However, the absence of prospective clinical data indicates that it would be premature to reach such a conclusion.

Going forward, prospective randomized trials of the two technologies should be carried out to further assess physician satisfaction with symmetry attained, degree of control of the spacing achieved, ease of use as well as acute and chronic toxicities experienced by men treated for prostate cancer. Only by meticulous assessment of the respective approaches will clinicians gain confidence with the use of spacer technologies to neutralize the dosimetric challenges created by the intricate anatomic relationship between the prostate and rectum.

## 5 CONCLUSION

This is the first comparative retrospective study to assess the dosimetry effectiveness of balloon spacer and SpaceOAR during prostate VMAT and UH treatments. Noteworthy rectal dose reduction greater than 75% was achieved in the post‐balloon spacer when using VMAT and UH treatment techniques. The use of balloon spacer significantly reduced the rectal dose regardless of the retrospective nature of this study.

Despite the expected superior advantages of the balloon spacer technique, a randomized prospective study is required to determine whether the balloon spacer will result also in a superior benefit for several clinical aspects of radiation including particle therapy, brachytherapy and post‐ prostatectomy setting for prostate radiotherapy patients.

## CONFLICT OF INTEREST STATEMENT

Michael Kos and Paulo Costa served as principle investigators and clinical proctors and were paid for each procedure as well as providing proctoring to other physicians clinical training.

## Data Availability

Research data are stored in an institutional repository and will be shared upon request to the corresponding author.
